# Real-life outcomes for oral disease-modifying treatments of relapsing-remitting multiple sclerosis patients: Adherence and adverse event profiles from Marmara University

**DOI:** 10.55730/1300-0144.5640

**Published:** 2023-02-01

**Authors:** Ezgi VURAL, Esin ENGİN, Gülin SÜNTER, Kadriye AĞAN, Dilek GÜNAL

**Affiliations:** Department of Neurology, School of Medicine, Marmara University, İstanbul, Turkey

**Keywords:** Multiple sclerosis, disease-modifying therapies, adverse event, treatment adherence, treatment drop-out

## Abstract

**Background/aim:**

Disease-modifying treatments (DMT) are used to prevent future relapses and disability. High long-term adherence to treatment is important to achieve disease control.

This study aims to investigate and compare adherence, adverse event (AE) profiles, and frequencies, main reasons for treatment discontinuation under Teriflunomide (TERI), Dimethyl Fumarate (DMF), and Fingolimod (FNG) for relapsing-remitting MS (RRMS) patients. This study is designed to explore patient-reported experiences in real-life settings.

**Materials and methods:**

Patients who were older than 18 years with a definite diagnosis of RRMS and no history of stem-cell transplantation were included. Outpatient clinic data files at the Neurology Department of Marmara University from June 2012 to June 2019 were examined retrospectively.

**Results:**

One hundred and ninety MS patients were enrolled. 118 FNG, 51 DMF, 44 TERI treatment cycles were recorded. Time since disease onset, time since diagnosis, and treatment duration were significantly longer for FNG (p = 0.012, p = 0.004, p < 0.001).

72.8% of all the treatment cycles were continued. There was no significant difference in treatment continuity between the 3 DMT groups. The most common reasons for treatment discontinuation in order of frequency were adverse events, the progression of the disease, and the persistence of relapses. No significant difference was found for treatment discontinuation reasons.

32% of the patients reported at least one AE. 28% FNG, 49 % DMF, and 27.3% TERI using patients reported AEs. AEs were much more frequently reported for DMF (p = 0.015). The most common adverse events for each DMT were alopecia (n = 6, 13.6%) for TERI, flushing for DMF (n = 20, 39.2%), and persistent lymphopenia for FNG (n = 9, 7.6%). No severe or life-threatening AE was reported for DMF, one patient experienced pancreatitis under TERI, and 11 (9.3%) patients using FNG had to stop treatment due to serious or life-threatening AEs including cardiac adverse events, opportunistic infections, and dysplasia.

**Conclusion:**

Overall treatment discontinuation because of AEs is as low as 10.3% in this study. However, AEs are still the main reason for treatment drop-out.

## 1. Introduction

Multiple sclerosis (MS) is a chronic demyelinating disease of the central nervous system. Treatment strategies for MS include treating acute relapses, applying disease-modifying treatments, and symptomatic treatments [1]. Disease-modifying treatments (DMT) vary in route and frequency of administration, adverse event profiles, and adherence, as well as aiming at different pathophysiological pathways [1]. DMTs should be offered to MS patients by their neurologists to prevent relapses and disease progression [2]. However, patients can only ensure benefit from DMTs by adhering to treatment and nonadherence is proven to increase relapse, as well as hospitalization rates for MS patients [3].

Adherence to treatment is referred to as obtaining prescriptions and administering treatment appropriately [4]. The most direct and easy way to monitor treatment adherence is by patients’ self-reports. So far, there are two studies investigating treatment adherence in MS patients in Turkey which mainly focused on the factors affecting treatment adherence and did not explore adherence and tolerability of orally administered DMTs [5,6].

Orally administered DMTs are easy for patients to use and high long-term adherence to treatment is crucial to achieve maximum disease control which seems to be a weak point for injectable therapies [7]. Three of the oral DMTs approved by the Ministry of Health in Turkey are Fingolimod (FNG), Dimethyl fumarate (DMF), and Teriflunomide (TERI). Tolerability and adverse event (AE) profiles for these 3 DMTs have been investigated in clinical trials [8–10].

This study aims to investigate and compare adherence, adverse event profiles, and frequencies, treatment outcomes, the main reasons for treatment discontinuation under either one of the mentioned oral DMTs for relapsing-remitting MS (RRMS) patients. This study is designed to explore patient-reported experiences in real-life settings.

## 2. Materials and methods

This is a retrospective and descriptive study conducted in a Turkish population with relapsing-remitting multiple sclerosis treated with oral disease-modifying therapies of teriflunomide (TERI), dimethyl fumarate (DMF), or fingolimod (FNG).

All treatment cycles were arranged following the approved label instructions. Treatment protocols involving TERI (14 mg once per day), DMF (120 mg twice per day for the first 7 days, then 240 mg twice per day), and FNG (0.5 mg once per day) are described elsewhere^1^,^2^,^3^.

Eligibility criteria were as followed: Age 18 years or older; a definite diagnosis of RRMS according to the McDonald criteria [11]; no history of stem-cell transplantation. Data files of MS outpatient clinic at the Neurology Department of Marmara University were examined retrospectively to identify patients who started TERI, DMF, or FNG between 1 June 2012 and 1 June 2019. Demographic (birth date, gender), clinical (date of disease onset, diagnosis date, disability score assessed by the Expanded Disability Status Scale [EDSS] [12], previous and present treatments, start and end dates of all treatments, main reasons for discontinuation, adverse events) data were recorded in the electronic hospital databases for all patients. Regardless of previous adverse event records, all the patients were phoned to be questioned about past and present possible adverse events.

To evaluate the treatment compliance and real-life safety of these drugs, we examined the frequency of AEs, treatment cessation rates, and the reasons behind it. An AE is defined as an undesirable medical occurrence in a subject after administration of a pharmaceutical product^4^. A serious AE was any AE which was life-threatening or caused death resulting in hospitalization, significant disability, or a congenital anomaly^5^. Lymphopenia was considered if the number of lymphocytes dropped below 200/mm^3^ under fingolimod treatment or if it persisted below 500/mm^3^ for 6 months under dimethyl fumarate treatment^6^ [13].

Data were analyzed using SPSS (Version 23.0, BM, New York, USA). Normal distribution analysis was conducted using the Kolmogorov-Smirnov test. Nonnormally distributed variables were compared using the Kruskal-Wallis test. Categorical variables were evaluated using the chi-square test. Categorical data were summarized by frequencies (percentages) and quantitative data were summarized as median (minimums and maximums). A p-value lower than 0.05 was considered significant.

Participants and their relatives were given clear information about the study and asked to read and sign the informed consent. Approval was obtained from the Ethics Committee of Marmara University, School of Medicine.

## 3. Results

A total of 190 MS patients were enrolled in the study and 213 total, 118 FNG, 51 DMF, and 44 TERI treatment cycles were recorded. 70.5% of all the patients were female and there was no statistically significant difference between TERI, DMF, and FNG patients concerning gender. Demographic and baseline clinical data are summarized in Table 1.

Patients under TERI were statistically significantly older (p = 0.001). Time since disease onset and time since diagnosis were found to be significantly longer for FNG using patients (p = 0.012 and p = 0.004). Moreover, the treatment duration for FNG was calculated to be significantly longer than TERI and DMF (p < 0.001). There was no statistically significant difference comparing EDSS scores at the baseline for each treatment (p = 0.5).

Most of the patients used more than one DMT at different times (Range 1–6). More patients under TERI or DMF treatment were treatment-naive, as TERI and DMF were chosen as an initial DMT, and they combined constitute 22.6% of the first-line agents. FNG was never a first treatment choice. Starting from second to fifth treatment choices FNG was the most chosen DMT among the orally taken DMTs.

72.8% of all the treatment cycles were continued. There was no significant difference in treatment continuity between the 3 DMT groups. The most common reasons for treatment discontinuation in order of frequency were adverse events, progression of the disease, and persistence of relapses. Only 3 treatment cycles were stopped due to patient nonadherence (Figure). Comparing the reasons for treatment discontinuation no significant difference was found for the 3 DMTs (Table 2). Thirty-two percent of the patients reported at least one adverse event. One hundred and two adverse events were recorded. Thirty-three (28%) FNG using patients reported 39 AEs, 25 (49%) patients reported 41 AEs for DMF, and 12 (27.3%) patients reported 22 AEs for TERI. Comparing the frequency of adverse events, DMF using patients reported AEs significantly much frequently than FNG and TERI using patients (p = 0.015). The most common adverse events for each DMT were as followed: alopecia (n = 6, 13.6%) for TERI, flushing for DMF (n = 20, 39.2%), persistent lymphopenia for FNG (n = 9, 7.6%). Detailed adverse event profiles are summarized in Tables 3, 4, and 5. No severe or life-threatening AE was reported for DMF, however, one patient experienced pancreatitis under TERI, and 11 (9.3%) patients using FNG had to stop treatment due to serious or life-threatening AEs. Of those 11 patients, 3 patients had cardiac adverse events, whereas 4 had opportunistic infections and 2 developed dysplasia.

## 4. Discussion

MS is known to affect women more commonly than men (ratio 2.3:1) [14]. 70.5% of whole patients in this study and more than 60% of patients in each treatment group were females.

Patients using TERI were statistically significantly older (p = 0.001). The mean age of MS patients in TEMSO (Study of Teriflunomide in Reducing the Frequency of Relapses and Accumulation of Disability in Patients With Multiple Sclerosis) trial ranged from 37.6 to 39.6 and the mean age of participants from all over the world except for the United States in TERI-Pro Phase 4 trial was 42.9 [8,15]. In line with data from previous studies regarding the mean age of patients under TERI treatment, the mean age to start TERI was 40.1 in our study. This is thought to be a result of possible teratogenic side-affects and clinicians refrain from starting TERI for women of childbearing age. Embryo-fetal toxicity and malformations in rats have been associated with teriflunomide exposure and because of that, it is contraindicated in pregnant women or women of childbearing potential who are not using reliable contraception^7^,^8^.

Twelve (27.3%) patients under TERI treatment reported 22 AEs. In this study, the most common AEs for TERI were alopecia (13.6%), abdominal pain (4.6%), menstrual irregularity (4.6%), and diarrhea (4.6%). Our findings differ from nine-year follow-up of the randomized TEMSO study which revealed that almost 90% of the participants reporting at least 1 AEs and the most common AEs being nasopharyngitis, headache, and alanine aminotransferase (ALT) increase [8]. Moreover, the incidence of any AE was as high as 84.6% in TERI-Pro Phase 4 trial and AE profile of our study seems to be confined to that study which indicated the most common AEs as hair thinning (31.2%), diarrhea (19.1%), and nausea (7.0%)[15]. There have been studies indicating less frequent AE under TERI, such as a Danish real-life study following 102 RRMS patients revealing AE incidence of 47% [16].

ALT increase was the most common reason for TERI discontinuation in extended TEMSO trial [8]. In our study only 1 patient discontinued TERI due to ALT increase and the others stopped treatment because of pancreatitis, generalized pain and one patient stopped treatment because of multiple AEs including menstrual irregularity, alopecia, migraine attacks.

Although mild to moderate infections could be expected, none of TERI using patients in our cohort reported infections. Hypertension in patients with no history of blood pressure irregularity or antihypertensive treatment was reported as an AE [8]. One patient experienced hypertension in our study.

According to a previous study, 10.6% of the patients discontinued TERI due to AEs. In the same study, the most common reasons for treatment discontinuation were adverse events (10.6%), lack of efficacy (5.3%), and other reasons (4.4%). Noncompliance frequency was as low as 1.1% and adherence to treatment was very high with 98.2% of patients reporting >80% compliance[17]. These treatment drop-out data are in accordance with our findings which indicated 9.1% of discontinuation because of AEs in our study group.

In TOWER (Efficacy Study of Teriflunomide in Participants with Relapsing Multiple Sclerosis) phase 3 trial incidence of serious AEs was 12% for 14 mg teriflunomide and in TEMSO extension trial serious AEs were reported by 20% of the patients. Compared to these previous phase 3 trials frequency of serious AEs was much lower in our study [8,18].

The safety and clinical efficacy of oral DMF were investigated in two randomized placebo-controlled phase III clinical trials, DEFINE (Efficacy and Safety of Oral BG00012 in Relapsing-Remitting Multiple Sclerosis) comparing DMF 240 mg twice daily and DMF 240 mg thrice daily with placebo and CONFIRM (Efficacy and Safety Study of Oral BG00012 with Active Reference in Relapsing-Remitting Multiple Sclerosis) comparing DMF 240 mg twice daily and DMF 240 mg thrice daily with glatiramer acetate in patients with RRMS [9,19]. The percentage of any AE for DMF 240 mg twice daily was 96% in DEFINE and 94% in CONFIRM. In our study, the frequency of any AE under DMF (49%) was lower compared to these studies.

DEFINE indicated the 3 most common AEs other than MS relapse to be flushing (38%), diarrhea (15%), nausea (13%), whereas the 3 most common AEs in CONFIRM study were flushing (31%), nasopharyngitis (17%) and headache (14%). In interim analysis of ENDORSE (BG00012 Monotherapy Safety and Efficacy Extension Study in Multiple Sclerosis) it was indicated that MS relapse and nasopharyngitis were most common in patients continuing DMF and flushing and GI-related events were more common among patients new to DMF [20]. Although our study did not investigate AE frequency regarding DMF start and continuity, our findings correlate with previous phase 3 trials, as the most common AEs were either skin-related or gastrointestinal (GI) related events. The most common AEs in our study were flushing (39%), itching (11.8%), rash (5.9%), abdominal pain (5.9%), and nausea (3.9%). None of our DMF using patients experienced a serious AE. However, it was shown that 18% of patients in DEFINE and 17% of patients in CONFIRM had a serious AE.

Time since disease onset and time since diagnosis were found to be longer for FNG using patients which might be the result of FNG’s approval as a second-line treatment agent by the Turkish Ministry of Health. Although FNG was administered later during the disease process, there was no disability difference between treatment groups. Besides, treatment duration for FNG was calculated to be significantly longer which could be speculated to be the consequence of FNG being a more potent DMT[21].

In this study, 28% of FNG using patients reported at least one AE and 9.3% of patients experienced a serious AE which led to treatment cessation. In the extension of randomized TRANSFORMS (Efficacy and Safety of Fingolimod in Patients With Relapsing-remitting Multiple Sclerosis With Optional Extension Phase) study the incidence of overall AEs was 94.7% and the frequency of overall serious AEs was 15.4%[13]. Although the percentage of patients reporting at least one AE seems to be lower in our study, the percentage of serious AE experiencing patients seems to be in line with TRANSFORMS study. However, real-life experiences might lead to different results than phase 3 trials. Analyzing 240 MS patients in the Czech Republic it was found that 35.0% of patients using fingolimod reported at least one AE and 4.2% had serious AEs[22]. In another study, 17.0% of 271 FNG using patients stopped treatment due to AEs[23].

The serious adverse events under FNG in our study group were cardiac events (tachycardia for 1 patient, chest tightness for 1 patient and bradycardia for 1 patient), opportunistic infections (for 4 patients) and dysplasia (dysplastic nevus for 1 patient and hyperplasia of intramammary lymph nodes for 1 patient). The most common serious AEs other than MS relapse in extended TRANSFORMS studies were basal cell carcinoma (1.7%), cholelithiasis (1.1%) and cystitis (0.6%) and breast cancer (0.6%) and in the previously mentioned Czech study, the AEs leading to discontinuation were macular edema, lymphopenia, liver transaminase elevation, hepatopathy, pain, and gynecological infection [13,22].

Although serious AE profiles seem to differ between studies the most common AEs share common features between different studies. In our study, the most common AEs under FNG treatment were persistent lymphopenia (7.6%), elevated liver enzymes (4.2%), abdominal pain (2.5%), and headache (1.7%). A German study investigating 36 months of real-world persistence and benefit-risk profile of FNG indicated the most common AEs as hypertension (2.1%), increased hepatic enzyme levels (2.0%), and increased ALT levels (0.7%) [24]. In extended TRANSFORMS study, the most common AEs were nasopharyngitis (31.5%), lymphopenia (21.9%), and headache (19.4%) [13].

Previous studies also compared AE frequency and profiles, as well as drop-out rates for oral DMTs. Significantly more frequently reported AEs (49%) under DMF treatment in this study correlates with the findings of a previous study which showed 26.5% of DMF patients and 12% of patients using TERI reported at least one AE (p < 0.001) [25]. Nevertheless, more frequent AEs did not affect DMF continuation in this study, there was no significant difference between the 3 DMT groups regarding treatment outcome. This finding in our study seems to be in contradiction with a previous study conducted on 395 DMF and 264 FNG patients assessing comparative efficacy and discontinuation. In that study, it was declared that DMF users were more likely to discontinue treatment because of intolerability [26]. Our findings regarding treatment discontinuation because of AEs seem to be in line with DEFINE (16%) and CONFIRM (12%).

High long-term adherence to treatment is crucial to achieve maximum disease control. Overall adherence to orally administered DMTs is high. In our study, only 1.4% of total treatment cycles ended due to nonadherence. In a population-based study, 11.0% of patients discontinued their initial oral DMT by 6 months and 19.6% discontinued their treatment by 12 months [27]. Noncompliance to injectable treatment options, which are commonly chosen as first-line agents, are reported to be 22.1% and in another study compliance rate to injection therapies was shown to be as low as 32.3% [7,28].

The main restriction of our study is the low number of patients. A low number of patients might influence the generalizability of our findings. In case of an increased number of participants treatment drop-out and AE rates might become more generalizable. Even though patients are questioned about possible AEs at every outpatient visit they were also phoned to be questioned about AEs they did not report and there is always a recall bias. Also, the cultural difference across the world might affect AE reporting. In the Turkish population, patients choose not to report an adverse event or even deny any AEs if those events are tolerable, or they vanish during the treatment process and if disease progression and relapses are well controlled. This might be one of the reasons for the low number of noncompliance and treatment drops out due to noncompliance in this study. Meanwhile, information regarding the most frequent AEs is shared with patients when evaluating possible treatment options. Patients are informed about temporary AEs and their foreseen duration. These factors might also increase the tolerability of AEs and decrease treatment drop-out due to AEs. We think that differences between different real-life studies investigating AEs might be affected by cultural backgrounds.

We did not examine the relationship between past treatments and reported adverse events. Even if recommended wash-out periods when switching treatments were followed, especially laboratory-based AEs such as lymphopenia might be affected by past treatment agents.

Finally, overall treatment discontinuation because of AEs is as low as 10.3%. However, AEs are still the main reason for treatment drop out. AEs should always be monitored, and it should be always kept in mind that serious and life-threatening AEs might be faced during the treatment process.

## Figures and Tables

**Figure:**
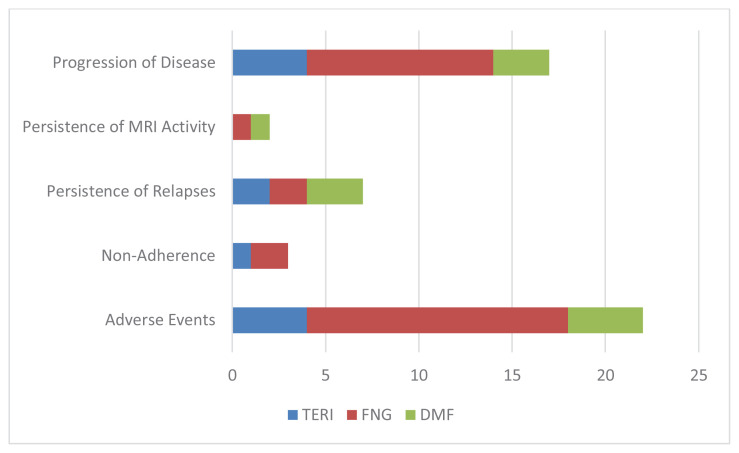
Distribution of the most common treatment discontinuation reasons in ended treatment cycles. TERI, teriflunomide; FNG, fingolimod; DMF, dimethyl fumarate

**Table 1:** Comparison of demographic and clinical baseline data between treatment groups.

	TERI	FNG	DMF	p Value
Male; n (%)	10 (22.7)	37 (31.4)	17 (33.3)	0.5
Female; n (%)	34 (77.3)	81 (68.6)	34 (66.7)	
Age at the treatment start; Median (min, max)	40.2 (18.5–65.2)	35 (19.8–59.3)	31.9 (19–58.4)	0.001
Time since diagnosis (years); Median (min, max)	3.6 (0.02–21)	6.7 (0.9–25)	4.1 (0.02–14.4)	0.004
Time since disease onset (years); Median (min, max)	3.9 (0.02–26.5)	7.2 (1–25)	6 (0.07–17.4)	0.01
EDSS; Median (min, max)	1.5 (1–6)	2 (1–7.5)	1.5 (1–6)	0.5

FNG, fingolimod; DMF, dimethyl fumarate; TERI, teriflunomide; EDSS, expanded disability status scale

**Table 2:** Comparison of treatment outcome and distribution of reasons for treatment discontinuation. n (%).

	TERI (n = 44)	FNG (n = 118)	DMF (n = 51)	Total (n = 213)	p
Treatment outcome					
Continued	33 (75)	83 (70.3)	39 (76.5)	155 (72.8)	0.7
Cessation	11 (25)	35 (29.7)	12 (23.5)	58 (27.2)	
Reason for treatment discontinuation					
Adverse event	4 (9.1)	14 (11.9)	4 (7.8)	22 (10.3)	0.9
Nonadherence	1 (2.3)	2 (1.7)	0 (0)	3 (1.4)	
Persistence of relapses	2 (4.6)	2 (1.7)	3 (5.9)	7 (3.3)	
Persisting MRI activity	0 (0)	1 (0.9)	1 (2)	2 (1)	
Pregnancy confirmed	0 (0)	3 (2.5)	0 (0)	3 (1.4)	
Pregnancy planning	0 (0)	1 (0.9)	1 (2)	2 (1)	
Progression of disease	4 (9.1)	10 (8.5)	3 (5.9)	17 (8)	

**Table 3:** Reported adverse events under fingolimod treatment n (%).

Adverse events	Lymphopenia	9 (7.6)
	Elevated liver enzymes	5 (4.2)
	Abdominal pain	3 (2.5)
	Headache	2 (1.7)
	Rash	2 (1.7)
	Hair loss	1 (0.9)
	Verricula	1 (0.9)
	Diarrhea	1 (0.9)
	Skin lesions	1 (0.9)
	Hypercoagulability	1 (0.9)
	Nasal congestion	1 (0.9)
Serious adverse events	Infection	3 (2.5)
	Periodontitis	1 (0.9)
	Tachycardia	1 (0.9)
	Bradycardia	1 (0.9)
	Hypertension	1 (0.9)
	Chest tightness	1 (0.9)
	Paresthesia	1 (0.9)
	Macular edema	1 (0.9)
	Dysplastic nevus	1 (0.9)
	Hyperplasia of intramammary lymph nodes	1 (0.9)

**Table 4:** Reported adverse events under dimethyl fumarate treatment n (%).

Adverse events	Flushing	20 (39.2)
	Itching	6 (11.8)
	Rash	3 (5.9)
	Abdominal pain	3 (5.9)
	Nausea	2 (3.9)
	Fatigue	2 (3.9)
	Musculoskeletal pain	1 (2)
	Lymphopenia	1 (2)
	Tinnitus	1 (2)
	Dyspepsia	1 (2)
	Vomiting	1 (2)

**Table 5:** Reported adverse events under teriflunomide treatment n (%).

Adverse events	Alopecia	6 (13.6)
	Abdominal pain	2 (4.6)
	Menstrual irregularity	2 (4.6)
	Diarrhea	2 (4.6)
	Hair thinning	1 (2.3)
	Abdominal distention	1 (2.3)
	Paresthesia	1 (2.3)
	Elevated liver enzymes	1 (2.3)
	Hair loss	1 (2.3)
	Generalized pain	1 (2.3)
	Hypertension	1 (2.3)
	Migraine	1 (2.3)
	Weight loss	1 (2.3)
Serious adverse events	Pancreatitis	1 (2.3)

## Footnotes

1 European Medicines Agency. Product Information, Aubagio (2013) [online]. Website https://www.ema.europa.eu/en/documents/product-information/aubagio-epar-product-information_en.pdf. [accessed Dec. 08, 2019].

2 European Medicines Agency. Product Information, Tecfidera (2014) [online] Website https://www.ema.europa.eu/en/documents/product-information/tecfidera-epar-product-information_en.pdf. [accessed Dec. 08, 2019].

3 European Medicines Agency. Product Information, Gilenya (2011) [online] Website https://www.ema.europa.eu/en/documents/product-information/gilenya-epar-product-information_en.pdf. [accessed Dec. 08, 2019].

4 European Medicines Agency. Guideline on good pharmacovigilance practices (GVP) - Module VI – Management and reporting of adverse reactions to medicinal products (Rev 1) (superseded) (2012) [online]. Website https://www.ema.europa.eu/en/documents/regulatory-procedural-guideline/guideline-good-pharmacovigilance-practices-gvp-module-vi-collection-management-submission-reports_en.pdf [accessed Dec. 08, 2019]

5 European Medicines Agency. Serious adverse reaction [online]. Website https://www.ema.europa.eu/en/glossary/serious-adverse-reaction [accessed Dec. 08, 2019].

6 European Medicines Agency. Updated recommendations to minimise the risk of the rare brain infection PML with Tecfidera (2015) [online]. Website https://www.ema.europa.eu/en/documents/press-release/updated-recommendations-minimise-risk-rare-brain-infection-pml-tecfidera_en.pdf. [accessed Dec. 08, 2019].

7 Teriflunomide mechanism of action: linking species’ sensitivities. ECTRIMS Online Library. (2015) [online]. Website https://onlinelibrary.ectrims-congress.eu/ectrims/2015/31st/115695/lynn.davenport.teriflunomide.mechanism.of.action.linking.species.sensitivities.html [accessed Dec. 08, 2019].

8 AUBAGIO^®^ (teriflunomide). Official Healthcare Professional Site. [online]. Website https://www.aubagiohcp.com/ [accessed Dec. 08, 2019].

## References

[1] Wiendl H Gold R Berger T Derfuss T Linker R Mäurer M Aktas O Baum K Berghoff M Bittner S Chan A Czaplinski A Deisenhammer F Di Pauli F Du Pasquier R Enzinger C Fertl E Gass A Gehring K Gobbi C Goebels N Guger M Haghikia A Hartung HP Heidenreich F Hoffmann O Kallmann B Kleinschnitz C Klotz L Leussink VI Leutmezer F Limmroth V Lünemann JD Lutterotti A Meuth SG Meyding-Lamadé U Platten M Rieckmann P Schmidt S Tumani H Weber F Weber MS Zettl UK Ziemssen T Zipp F ‘Multiple Sclerosis Therapy Consensus Group’ (MSTCG) Multiple Sclerosis Therapy Consensus Group (MSTCG): position statement on disease-modifying therapies for multiple sclerosis (white paper) Ther Adv Neurol Disord 2021 Aug 18 14 17562864211039648 https://doi:10.1177/17562864211039648 34422112 10.1177/17562864211039648 PMC8377320

[2] Montalban X Gold R Thompson AJ Otero-Romero S Amato MP Chandraratna D Clanet M Comi G Derfuss T Fazekas F Hartung HP Havrdova E Hemmer B Kappos L Liblau R Lubetzki C Marcus E Miller DH Olsson T Pilling S Selmaj K Siva A Sorensen PS Sormani MP Thalheim C Wiendl H Zipp F ECTRIMS/EAN Guideline on the pharmacological treatment of people with multiple sclerosis Mult Scler 2018 Feb 24 2 96 120 https://doi:10.1177/1352458517751049 29353550 10.1177/1352458517751049

[3] Steinberg SC Faris RJ Chang CF Chan A Tankersley MA Impact of adherence to interferons in the treatment of multiple sclerosis: a non-experimental, retrospective, cohort study Clin Drug Investig 2010 30 2 89 100 10.2165/11533330-000000000-00000 20067327

[4] Kołtuniuk A Pytel A Krówczyńska D Chojdak-Łukasiewicz J The Quality of Life and Medication Adherence in Patients with Multiple Sclerosis-Cross-Sectional Study Int J Environ Res Public Health 2022 Nov 5 19 21 14549 10.3390/ijerph192114549 36361427 PMC9656792

[5] Köşkderelioğlu A Gedizlioğlu M Ortan P Öcek Ö Evaluation of the Adherence to Immunmodulatory Treatment in Patients with Multiple Sclerosis Noro Psikiyatr Ars 2015 Dec 52 4 376 379 10.5152/npa.2015.8825 28360743 PMC5353111

[6] Erbay Ö Usta Yeşilbalkan Ö Yüceyar N Factors Affecting the Adherence to Disease-Modifying Therapy in Patients With Multiple Sclerosis J Neurosci Nurs 2018 Oct 50 5 291 297 10.1097/JNN.0000000000000395 30138155

[7] Hansen K Schüssel K Kieble M Werning J Schulz M Friis R Pöhlau D Schmitz N Kugler J Adherence to Disease Modifying Drugs among Patients with Multiple Sclerosis in Germany: A Retrospective Cohort Study PLoS One 2015 Jul 27 10 7 e0133279 10.1371/journal.pone.0133279 26214805 PMC4516264

[8] O’Connor P Comi G Freedman MS Miller AE Kappos L Bouchard JP Lebrun-Frenay C Mares J Benamor M Thangavelu K Liang J Truffinet P Lawson VJ Wolinsky JS Teriflunomide Multiple Sclerosis Oral (TEMSO) Trial Group and the MRI-AC in Houston, Texas Long-term safety and efficacy of teriflunomide: Nine-year follow-up of the randomized TEMSO study Neurology 2016 Mar 8 86 10 920 30 10.1212/WNL.0000000000002441 26865517 PMC4782117

[9] Gold R Kappos L Arnold DL Bar-Or A Giovannoni G Selmaj K Tornatore C Sweetser MT Yang M Sheikh SI Dawson KT DEFINE Study Investigators Placebo-controlled phase 3 study of oral BG-12 for relapsing multiple sclerosis N Engl J Med 2012 Sep 20 367 12 1098 107 10.1056/NEJMoa1114287 22992073

[10] Kappos L Radue EW O’Connor P Polman C Hohlfeld R Calabresi P Selmaj K Agoropoulou C Leyk M Zhang-Auberson L Burtin P FREEDOMS Study Group A placebo-controlled trial of oral fingolimod in relapsing multiple sclerosis N Engl J Med 2010 Feb 4 362 5 387 401 10.1056/NEJMoa0909494 20089952

[11] Thompson AJ Banwell BL Barkhof F Carroll WM Coetzee T Comi G Correale J Fazekas F Filippi M Freedman MS Fujihara K Galetta SL Hartung HP Kappos L Lublin FD Marrie RA Miller AE Miller DH Montalban X Mowry EM Sorensen PS Tintoré M Traboulsee AL Trojano M Uitdehaag BMJ Vukusic S Waubant E Weinshenker BG Reingold SC Cohen JA Diagnosis of multiple sclerosis: 2017 revisions of the McDonald criteria Lancet Neurol 2018 Feb 17 2 162 173 10.1016/S1474-4422(17)30470-2 29275977

[12] Kurtzke JF Rating neurologic impairment in multiple sclerosis: an expanded disability status scale (EDSS) Neurology 1983 Nov 33 11 1444 52 10.1212/wnl.33.11.1444 6685237

[13] Cohen JA Khatri B Barkhof F Comi G Hartung HP Montalban X Pelletier J Stites T Ritter S von Rosenstiel P Tomic D Kappos L TRANSFORMS (TRial Assessing injectable interferoN vS. FTY720 Oral in RRMS) Study Group Long-term (up to 4.5 years) treatment with fingolimod in multiple sclerosis: results from the extension of the randomised TRANSFORMS study J Neurol Neurosurg Psychiatry 2016 May 87 5 468 75 10.1136/jnnp-2015-310597 26111826 PMC4853559

[14] Alonso A Hernán MA Temporal trends in the incidence of multiple sclerosis: a systematic review Neurology 2008 Jul 8 71 2 129 35 10.1212/01.wnl.0000316802.35974.34 18606967 PMC4109189

[15] Coyle PK Khatri B Edwards KR Meca-Lallana JE Cavalier S Rufi P Benamor M Poole EM Robinson M Gold R Teriflunomide real-world evidence: Global differences in the phase 4 Teri-PRO study Mult Scler Relat Disord 2019 Jun 31 157 164 10.1016/j.msard.2019.03.022 31005729

[16] Elkjaer ML Molnar T Illes Z Teriflunomide for multiple sclerosis in real-world setting Acta Neurol Scand 2017 Nov 136 5 447 453 10.1111/ane.12755 28321835

[17] Moccia M Palladino R Carotenuto A Russo CV Triassi M Lanzillo R Brescia Morra V Predictors of long-term interferon discontinuation in newly diagnosed relapsing multiple sclerosis Mult Scler Relat Disord 2016 Nov 10 90 96 10.1016/j.msard.2016.09.011 27919507

[18] Confavreux C O’Connor P Comi G Freedman MS Miller AE Olsson TP Wolinsky JS Bagulho T Delhay JL Dukovic D Truffinet P Kappos L TOWER Trial Group Oral teriflunomide for patients with relapsing multiple sclerosis (TOWER): a randomised, double-blind, placebo-controlled, phase 3 trial Lancet Neurol 2014 Mar 13 3 247 56 10.1016/S1474-4422(13)70308-9 24461574

[19] Fox RJ Miller DH Phillips JT Hutchinson M Havrdova E Kita M Yang M Raghupathi K Novas M Sweetser MT Viglietta V Dawson KT CONFIRM Study Investigators Placebo-controlled phase 3 study of oral BG-12 or glatiramer in multiple sclerosis N Engl J Med 2012 Sep 20 367 12 1087 97 10.1056/NEJMoa1206328 22992072

[20] Gold R Arnold DL Bar-Or A Hutchinson M Kappos L Havrdova E MacManus DG Yousry TA Pozzilli C Selmaj K Sweetser MT Zhang R Yang M Potts J Novas M Miller DH Kurukulasuriya NC Fox RJ Phillips TJ Long-term effects of delayed-release dimethyl fumarate in multiple sclerosis: Interim analysis of ENDORSE, a randomized extension study Mult Scler 2017 Feb 23 2 253 265 10.1177/1352458516649037 27207449 PMC5418934

[21] Prosperini L Lucchini M Haggiag S Bellantonio P Bianco A Buscarinu MC Buttari F Centonze D Cortese A De Giglio L Fantozzi R Ferraro E Fornasiero A Francia A Galgani S Gasperini C Marfia GA Millefiorini E Nociti V Pontecorvo S Pozzilli C Ruggieri S Salvetti M Sgarlata E Mirabella M Fingolimod vs dimethyl fumarate in multiple sclerosis: A real-world propensity score-matched study Neurology 2018 Jul 10 91 2 e153 e161 10.1212/WNL.0000000000005772 29875218

[22] Tichá V Kodým R Počíková Z Kadlecová P Real-World Outcomes in Fingolimod-Treated Patients with Multiple Sclerosis in the Czech Republic: Results from the 12-Month GOLEMS Study Clin Drug Investig 2017 Feb 37 2 175 186 10.1007/s40261-016-0471-2 PMC5250638 27785735

[23] Vollmer B Nair KV Sillau SH Corboy J Vollmer T Alvarez E Comparison of fingolimod and dimethyl fumarate in the treatment of multiple sclerosis: Two-year experience Mult Scler J Exp Transl Clin 2017 Aug 17 3 3 2055217317725102 10.1177/2055217317725102 28839949 PMC5564884

[24] Ziemssen T Lang M Tackenberg B Schmidt S Albrecht H Klotz L Haas J Lassek C Couto CA Findlay JA Cornelissen C PANGAEA study group Real-world persistence and benefit-risk profile of fingolimod over 36 months in Germany Neurol Neuroimmunol Neuroinflamm 2019 Mar 7 6 3 e548 10.1212/NXI.0000000000000548 30882022 PMC6410931

[25] D’Amico E Zanghì A Callari G Borriello G Gallo A Graziano G Valentino P Buccafusca M Cottone S Salemi G Ragonese P Bossio RB Docimo R Grimaldi LME Pozzilli C Tedeschi G Zappia M Patti F Comparable efficacy and safety of dimethyl fumarate and teriflunomide treatment in Relapsing-Remitting Multiple Sclerosis: an Italian real-word multicenter experience Ther Adv Neurol Disord 2018 Sep 10 11 1756286418796404 10.1177/1756286418796404 30210582 PMC6131312

[26] Hersh CM Love TE Bandyopadhyay A Cohn S Hara-Cleaver C Bermel RA Fox RJ Cohen JA Ontaneda D Comparative efficacy and discontinuation of dimethyl fumarate and fingolimod in clinical practice at 24-month follow-up Mult Scler J Exp Transl Clin 2017 Aug 24 3 3 2055217317715485 10.1177/2055217317715485 28890796 PMC5574489

[27] Setayeshgar S Kingwell E Zhu F Zhang T Carruthers R Marrie RA Evans C Tremlett H Persistence and adherence to the new oral disease-modifying therapies for multiple sclerosis: A population-based study Mult Scler Relat Disord 2019 Jan 27 364 369 10.1016/j.msard.2018.11.004 30476872

[28] McKay KA Tremlett H Patten SB Fisk JD Evans C Fiest K Campbell T Marrie RA CIHR Team in the Epidemiology and Impact of Comorbidity on Multiple Sclerosis (ECoMS) Determinants of non-adherence to disease-modifying therapies in multiple sclerosis: A cross-Canada prospective study Mult Scler 2017 Apr 23 4 588 596 10.1177/1352458516657440 27357507 PMC5407504

